# Characterization of Enzyme-Linked Immunosorbent Assay (ELISA) for Quantification of Antibodies against *Salmonella* Typhimurium and *Salmonella* Enteritidis O-Antigens in Human Sera

**DOI:** 10.3390/biotech12030054

**Published:** 2023-08-11

**Authors:** Maria Grazia Aruta, Elisa Lari, Daniele De Simone, Bianca Semplici, Claudia Semplici, Helen Dale, Esmelda Chirwa, Innocent Kadwala, Maurice Mbewe, Happy Banda, Miren Iturriza-Gomara, Melita Gordon, Tonney Nyirenda, Pietro Piu, Mariagrazia Pizza, Francesco Berlanda Scorza, Silvia Grappi, Rocío Canals, Omar Rossi, on behalf of the Vacc-iNTS Consortium Collaborators

**Affiliations:** 1 GSK Vaccines Institute for Global Health (GVGH) S.r.l., 53100 Siena, Italy; maria-grazia.x.aruta@gsk.com (M.G.A.); daniele.x.desimone@gsk.com (D.D.S.); miren.x.iturriza@gsk.com (M.I.-G.); m.pizza@imperial.ac.uk (M.P.); francesco.x.berlandascorza@gsk.com (F.B.S.);; 2 VisMederi S.r.l., 53100 Siena, Italy; elisa.lari@vismederi.com (E.L.); bianca.semplici@vismederi.com (B.S.); claudia.semplici@vismederi.com (C.S.); pietro.piu@vismederi.com (P.P.); silvia.grappi@vismederi.com (S.G.); 3 Malawi Liverpool Wellcome Trust Programme, Blantyre 30096, Malawi; helen.dale@liverpool.ac.uk (H.D.); echirwa@mlw.mw (E.C.); ikadwala@mlw.mw (I.K.); mombewe@mlw.mw (M.M.); hbanda@mlw.mw (H.B.); magordon@liverpool.ac.uk (M.G.); 4 Pathology Department, Kamuzu University of Health Sciences, Blantyre 312225, Malawi; tnyirenda@kuhes.ac.mw; 5 Institute of Infection, Veterinary and Ecological Sciences, University of Liverpool, Liverpool L69 3BX, UK; 6 Imperial College South Kensington Campus, London SW7 2AZ, UK

**Keywords:** Generalized Modules for Membrane Antigens (GMMA), Enzyme-Linked Immunosorbent Assay (ELISA), non-typhoidal *Salmonella*, human sera, antibodies, vaccine, iNTS

## Abstract

Nontyphoidal Salmonella (NTS) is a leading cause of morbidity and mortality caused by enteric pathogens worldwide in both children and adults, and vaccines are not yet available. The measurement of antigen-specific antibodies in the sera of vaccinated or convalescent individuals is crucial to understand the incidence of disease and the immunogenicity of vaccine candidates. A solid and standardized assay used to determine the level of specific anti-antigens IgG is therefore of paramount importance. In this work, we presented the characterization of a customized enzyme-linked immunosorbent assay (ELISA) with continuous readouts and a standardized definition of EU/mL. We assessed various performance parameters: standard curve accuracy, dilutional linearity, intermediate precision, specificity, limits of blanks, and quantification. The simplicity of the assay, its high sensitivity and specificity coupled with its low cost and the use of basic consumables and instruments without the need of high automation makes it suitable for transfer and application to different laboratories, including resource-limiting settings where the disease is endemic. This ELISA is, therefore, fit for purpose to be used for quantification of antibodies against *Salmonella* Typhimurium and *Salmonella* Enteritidis O-antigens in human samples, both for vaccine clinical trials and large sero-epidemiological studies.

## 1. Introduction

Worldwide, *Salmonella* Typhimurium and *Salmonella* Enteritidis are leading causative agents of foodborne illness in both children and adults [1]. Emerging lineages of *Salmonella enterica* serovars Typhimurium and Enteritidis have been associated with invasive non-typhoidal *Salmonella* (iNTS) disease, especially in sub-Saharan Africa. In this area, iNTS disease represents one of the major causes of morbidity and mortality, resulting in about 67,000 deaths each year [2]. Four risk factors associated with the prevalence of iNTS (malaria, HIV, malnutrition, and access to improved drinking water sources) have been taken into consideration in order to generate the iNTS risk factor (iNRF) index. An evaluation based on the iNRF index has shown that the risk level of iNTS varies among different geographies in sub-Saharan Africa, not only at the country level but also within the same country [3]. Nontyphoidal *Salmonella* (NTS) isolates in these resource-limited settings, where routine surveillance for antimicrobial resistance is rare, have been associated with multidrug resistance (MDR) [4].

The development of a vaccine against iNTS disease is becoming an urgent need in endemic areas, as no licensed vaccines to prevent this disease are currently available [5]. Several vaccine candidates are under development based on various technologies, both traditional and innovative [6]. All the approaches have a common denominator, which is the assumption that the key drivers of immunity against nontyphoidal *Salmonella*, as for other Gram-negative bacteria, are outer membrane surface antigens, with a particular focus on surface polysaccharides. One of the most advanced vaccine candidates, currently in clinical development, includes NTS components consisting of glycoconjugates of lipopolysaccharide-derived core and O-polysaccharide (COPS) linked to FliC flagellin [7]. A different strategy to deliver O-antigen polysaccharides uses the Generalized Modules for Membrane Antigens (GMMA) technology. A bivalent vaccine candidate of *Salmonella* Typhimurium and *Salmonella* Enteritidis GMMA is currently in clinical phase I [8,9,10,11]. GMMA are exosomes naturally released from Gram-negative bacteria that have been engineered to disrupt the linkage between the inner and the outer membrane generating a hyper-blebbing phenotype [12] and to deacylate the lipid A of the lipopolysaccharide reducing the risk of systemic reactogenicity when delivered parenterally [9]. The use of GMMA technology allows us to present surface polysaccharides and outer membrane proteins in their native conformation [12,13]. Furthermore, GMMA have self-adjuvanting properties, likely because they naturally contain various pathogen-associated molecular pattern molecules, because of their size, and their potential to present multiple antigens in their native conformation which is optimal to induce strong immunogenicity [13]. Since GMMA technology involves a relatively simple production process without the need for complex conjugation, it represents an attractive and affordable option particularly relevant for low- and middle-income countries [10,12]. Preclinical results have supported the transition of an iNTS GMMA-based vaccine into clinical development. In a comparison between iNTS GMMA and glycoconjugates, GMMA showed superiority to classical conjugates when tested in mice in terms of antibody quality and functionality [10].

*Salmonella* infections show a complex pathogenesis that consists of an intracellular antibody-refractive growth phase and an extracellular antibody-susceptible phase of spread [14]. Therefore, the measurement of antigen-specific antibodies in the sera of vaccinated or convalescent individuals could be fundamental to understanding the incidence of disease and the potential efficacy of vaccination. Traditionally, the method of choice to evaluate the level of specific anti-antigens IgG is the Enzyme-Linked Immunosorbent Assay (ELISA), which has been extensively used to test the response to vaccine antigens, both polysaccharides, and proteins, of several bacterial pathogens [14,15,16,17,18,19].

Various ELISA methods have been developed over the years to measure antibodies in samples including serum, plasma, urine, or feces [20]. Essentially, there are two major types of assays: titer-based with discrete readout, and assays based on continuous readout which rely on calibrated standard curves. The advantages of the titer-based approach are the simplicity of the assay and the fact that it is not mandatory for the setup of a calibrated standard serum, simplifying the effort when large studies need to be performed in multiple laboratories. However, a weakness is the discrete readout, and, thus, the difficulty to fully assess and compare the performances among different laboratories and between runs. In contrast, the assays based on continuous readout have the advantage of offering the possibility to measure the concentration of antibodies against a calibrated standard curve. These assays are the most commonly used as they allow us to compare results among laboratories and between runs by relying on calibrated standard curves in each plate. Results in continuous readout are expressed as antibody concentration (μg/mL), arbitrary ELISA Unit (EU/mL), or international units (IU/mL) for assays calibrated against international standards. A disadvantage of these assays is the need for a representative standard sera, whose availability can represent a bottleneck with the resulting need to put in place systems to bridge secondary standards. In terms of throughput, currently both titers-based and assays-based in continuous readout are similar with different levels of automation in terms of sample handling and data analysis allowing to speed up substantially the data generation and elaboration.

Here, we present the intra-laboratory characterization of a customized ELISA assay to determine anti-*S*. Typhimurium O-antigen and anti-*S*. Enteritidis O-antigen total IgG in human sera. We have characterized the assay determining standard curve accuracy, dilutional linearity, repeatability (intra-day precision), intermediate precision (inter-day precision), and specificity and have determined the limit of blanks and qualifications in addition to a series of solid quality control acceptance criteria.

## 2. Materials and Methods

### 2.1. Reagents

Phosphate Buffer Saline at pH 7 (PBS) was used for the preparation of different buffers: PBS + milk 5% (by adding 5% fat-free milk, Sigma, to PBS), washing buffer–PBS-T—(by adding 0.05% Tween-20) and secondary antibody buffer by adding 0.1% BSA (Sigma) to PBS-T. The coating buffer is a 0.05 M carbonate buffer, pH 9.6 (Sigma-Aldrich). Anti-human IgG-alkaline Phosphatase (Sigma cod. A3187) was used as the secondary antibody. The O-Antigens (OAg) used as coating antigens were extracted from GMMA purified from *S.* Typhimurium (STm) and *S.* Enteritidis (SEn) Δ*tolR*Δ*pagP*Δ*msbB* strains [9], by direct acid hydrolysis; polysaccharides were fully characterized analytically in terms of sugar content, O-acetylation level, protein, and nucleic acid impurities as previously reported [21]. OAg aliquotes for *S.* Typhimurium and *S.* Enteritidis were stored at −80 until use. The main OAg population for both coatings was at a molecular size of about 30 kDa, with protein impurities < 1% and nucleic acid impurities < 10 ng/mL.

### 2.2. ELISA Procedure and Calculation

Anti-STm OAg and anti-SEn OAg specific total IgG are measured in sera samples using *S*. Typhimurium and *S*. Enteritidis OAg as coating antigens at a final concentration of 5 µg/mL or 15 µg/mL, respectively, following the below protocol: coating overnight (16 h) at 4 °C of NuncMaxisorp 96-well round bottom (Nunc) plates, followed by aspiration (without wash) and blocking with 5% PBS milk for 1 h at 25 °C; washing of plates 3 times with PBS-T, before addition of primary antibodies (sera samples) diluted in 5% PBS milk, incubated for 2 h at 25 °C. Each human serum sample was run in triplicate at different dilutions (1:100, 1:4000, and 1:160,000, respectively) in PBS milk 5%. Plates are then washed 3 times with PBS-T and incubated for 1 h at 25 °C with secondary antibodies diluted 1:5000 in PBS-T plus 0.1% BSA, before the final 3 washes with PBS-T and addition of p-Nitrophenyl phosphate substrate (Sigma-fast, Sigma-Aldrich, Massachusetts, United States) for 1 h at 25 °C. Absorbances were read with a spectrophotometer at 405 and 490 nm (Biotek automatic plate reader), maintaining strict timing between plates. 

ELISA units are expressed in relation to a five-parameter human antigen-specific antibody standard serum curve composed of 10 standard points and 2 blank wells (run in duplicate on each plate). One ELISA unit is defined as the reciprocal of the dilution of the standard serum that gives an absorbance value (optical density measured at 405 nm subtracted to optical density measured at 490 nm—the latter being the background wavelength of the plastic of the plate) equal to 1. High control and low control (HC and LC, respectively) were run on each plate at dilution able to give as result a range equivalent to 1.3–2.8 EU/mL, respectively, for LC and HC. 

The primary anti-*Salmonella* standard sera was calibrated against each coating antigen, and antigen concentration was set at saturation of the signal for each antigen. 

Several QC criteria were applied on each run, in particular, the R-square value for the 5 PL curve fit of the standard dilution series (for both STm and SEn), maximum background OD, the minimum value of OD maximum, range of acceptance in terms of OD for 1 EU/mL (in case of SEn, deviation to the expected EU/mL both for high and low controls). If at least one of the above-mentioned criteria was not met, the entire layout was repeated under the same experimental conditions. A sample is instead considered valid if the EU/mL determined as average from the values obtained in the three independent plates have a CV% < 30% at the dilution selected for obtaining results (the ones in which OD values obtained fall within the linear part of the standard curve); if not met, the sample was re-run under the same assay conditions. 

### 2.3. Ethical Statement

The human serum pool used in this study was derived from Malawian healthy donors originally enrolled in the STRATAA (Strategic Typhoid Alliance across Africa and Asia) epidemiological study [22]. The relevant ethical and regulatory approval was obtained from the respective institutional and national ethics review committees (National Health Sciences Research Committee approval # 15/11/1511). Written informed consent was obtained before enrollment from all subjects and the trial was designed and conducted in accordance with the Good Clinical Practice Guidelines and the Declaration of Helsinki.

### 2.4. Serum Samples

iNTS Primary Human Standard Serum has been generated by Malawi-Liverpool-Wellcome Trust Clinical Research Programme (MLW) by pooling sera from highly positive Malawian adult subjects who were originally enrolled in a community-based randomly selected cohort within the STRATAA [22] epidemiological study. Forty positive (20 against STm OAg and 20 against SEn OAg) human sera from adults naturally exposed to iNTS were also used to characterize the assay. Working aliquots of the standard serum and the human single sera were stored at −80 °C until use. 

Various aliquots of iNTS Primary Human Standard Serum and the 40 human single sera were used and treated as described below to determine the different assay parameters. Human IgG-depleted serum (Molecular Innovations cod. HPLA-SER-GF) was used as a negative matrix. 

Samples used to assess standard curve accuracy: iNTS Primary Human Standard Serum has been used to prepare 24 standard curves, each composed of ten 2-fold dilutions of a curve at 10 EU/mL and 2 blanks.

Samples used to assess precision, and the lower and upper limit of quantification: 40 human single sera from adults subjects (20 previously screened for positivity against STm OAg and 20 against SEn OAg) were assayed independently by two operators working on the same days, in two independent replicates on each plate, on three different days (12 measurements in total for each individual serum).

Samples to assess linearity: iNTS Primary Human Standard Serum was assayed neat or diluted 1/2, 1/4, 1/8, 1/16, 1/32, 1/64, 1/128, and 1/256 in negative matrix prior to probing it as a sample in the assay.

Samples to assess specificity: two high responders (>500 EU/mL in the assay) human sera for STm and two high responders human sera for SEn, prediluted 1:50 in PBS + 5% milk, were preincubated overnight at 4 °C with an equal volume of homologous competitor at the final concentrations of 250, 50, 20, 5, and 1 µg/mL in PBS + 5% milk, in comparison to sera diluted overnight 1:100 in PBS + 5% milk (negative control). The lowest concentration of OAg able to cause a reduction of the ELISA Units ≥ 80% was then used to determine the homologous (in the presence of STm OAg for STm specificity and SEn OAg for SEn specificity) and heterologous specificity, assessed with samples incubated with OAg from a different species (Shigella flexneri 3a OAg) in comparison to undepleted control. All samples were incubated overnight (16–18 h) at 4 °C prior to being tested.

### 2.5. Statistical Analysis

Test results have been analyzed using Excel and GraphPad PRISM software version 7. Geometric and arithmetic mean, standard deviation, and coefficient of variation are the major statistics. To support the assessment of linearity, a log-log regression model was applied to measure the sensitivity of the response (i.e., the geometric mean of the test results) to the dilution levels. The limits of standard curve accuracy were calculated by linear interpolation. The precision results (CVs%) were resampled 1000 times by the bootstrap method, and the geometric mean was calculated at each iteration, obtaining the distribution of geometric means. The expected geometric mean was the geometric mean of the distribution. The lower limit of 95% confidence intervals (CI) was obtained as the quantile that divides the data distribution, leaving 2.5% of the distribution to its left, and the upper was obtained as the quantile that divides the data distribution leaving 2.5% of the distribution to its right.

## 3. Results

### 3.1. Experimental Method and Controls Setup 

**Figure 1:**
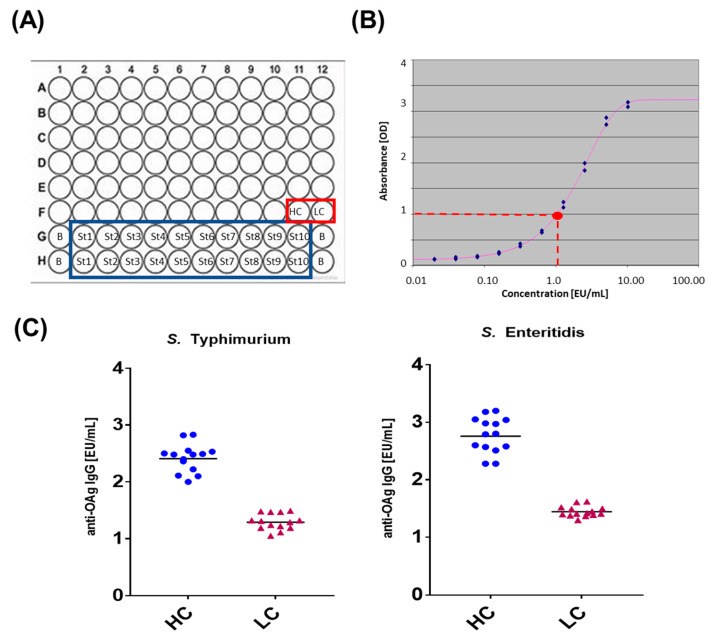
(**A**) Layout of a 96-well plate used in ELISA assay allowing analysis of 70 different sera (A1-F10); high and low control (HC and LC, respectively) highlighted by red rectangle (positions F11-F12); standard curve in 10 serial dilution points (ST1 to ST10) in duplicate highlighted by blue rectangle and four blank wells (**B**) (G1, H1, G12, H12); (**B**) a representative ELISA standard curve obtained by fitting a 5 parameter logistic (5 PL) curve to the individual values obtained at different experimental dilutions tested for the curve. One ELISA unit is defined as the reciprocal of the dilution of the standard serum that gives an absorbance value equal to 1 (red circle); (**C**) high control and low control against both serotypes have been set as the average value of 12 replicates.

Understanding the level of antibodies generated against specific antigens upon natural exposure to a pathogen is one of the drivers to evaluate the response, and infer potential protection of a vaccine against the same targets. Besides the complexity of nontyphoidal *Salmonella* (NTS) infection, the importance of antibodies elicited against the O-antigen portion of the lipopolysaccharide in NTS has been reported [14] and the most advanced vaccines are targeting the O-antigen. To perform a fully quantitative assessment of O-antigen-specific antibodies both from clinical trials and sero-epidemiological studies, it is essential to have a highly sensitive, efficient, and versatile assay. To this aim, we set up and characterized an ELISA to determine the level of IgG elicited against *Salmonella* Typhimurium and *Salmonella* Enteritidis O-antigens in human samples. In this assay, one ELISA unit is defined as the reciprocal of the dilution of the standard serum that gives an absorbance value equal to 1. High control and low control (HC and LC, respectively—Figure 1C) are run on each plate together with a calibrated standard curve (Figure 1B) composed of 10 serial dilution points in duplicate and 4 blank wells. Individual samples are tested in up to three dilutions, each run in triplicates, in different plates. EU/mL are therefore obtained by interpolating the OD values against an antigen-specific standard curve run on each plate. By using this method up to 70 different sera can be assayed on a set of 96-well plates (a “layout”, Figure 1A). The setup plates are composed of up to nine 96-well plates, three in which each individual sample is tested at the dilution 1:100, three in which each individual sample is tested at the dilution 1:4000, and three in which each individual sample is tested at the dilution 1:160,000. 

To be valid, an assay must pass several non-mutually exclusive quality control criteria both for the standard curve and controls (Table 1). If only one of those criteria is not met, the entire layout must be repeated under the same experimental conditions. A sample is instead valid if the EU/mL determined as average from the values obtained in the three independent plates have a CV% < 30% at the selected dilution.

The primary anti-*Salmonella* standard sera was calibrated against each coating antigen, and antigen concentration was set at saturation of the signal for each antigen.

The assay was characterized in terms of standard curve accuracy, precision, dilutional linearity, and specificity. As samples, ad hoc dilutions of the standard sera or sera (20 against STm and 20 against SEn) from naturally exposed individuals were used.

### 3.2. Standard Curve Accuracy 

**Figure 2:**
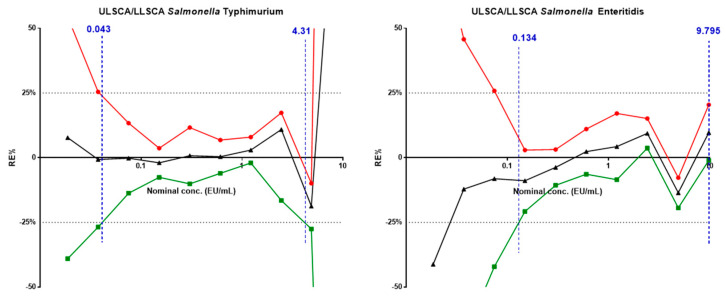
Upper and Lower Limit Standard Curve Accuracy (ULSCA/LLSCA). Average RE% (black line) and respective upper (red line) and lower (green line) 90% confidence interval for each standard curve point have been plotted in function of nominal concentration. Black horizontal dashed lines represent the acceptance range [+25%; −25%] of RE%. Vertical blue dashed lines represent the values of LLSCA and ULSCA.

Lower and upper limits of standard curve accuracy (LLSCA and ULSCA) represent, respectively, the lowest and the highest concentration of analyte based on the standard curve that can be quantitatively measured with suitable accuracy under assay conditions. To evaluate standard curve accuracy, 24 independent replicates of the standard curve were run in a standard assay. For each of the antigens, limits of standard curve accuracy were calculated as equivalent to the last and the first datapoints, respectively, at which the confidence interval of residual error percentage (RE%) felt within the acceptance range of [−25%; +25%] with 90% probability (Figure 2).

Values of LLSCA and ULSCA resulted to be 0.043 EU/mL and 4.313 EU/mL for *S*. Typhimurium and 0.134 EU/mL and 9.795 EU/mL for *S*. Enteritidis, respectively. The EU/mL of a specific sample is, therefore, calculated if the reading value falls within the above-reported accuracy range of the standard curve. Actual EU/mL for specific samples are subsequently calculated by multiplying the corresponding dilution from which EU/mL in the well were retrieved.

### 3.3. Linearity

The linearity of an analytical procedure is defined as the ability of the method to obtain, within a given range, test results that are directly proportional to the concentration of the analyte being measured. To evaluate the linearity, standard serum was tested at nine independent dilutions (neat, 1/2, 1/4, 1/8, 1/16, 1/32, 1/64, 1/128, and 1/256), and each dilution was prepared two times independently (for each coating antigen) by two operators, on three different days, resulting in twelve independent measurements in total for each dilution tested.

**Figure 3:**
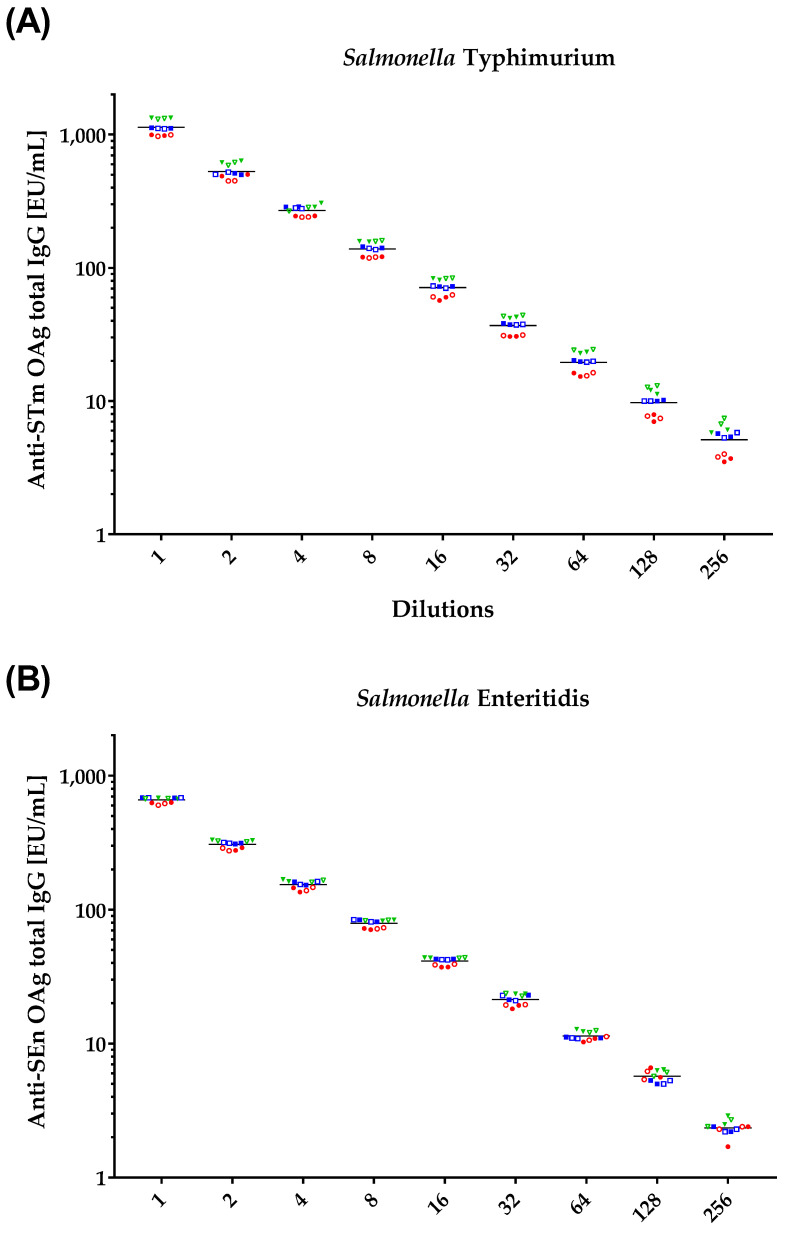
Anti-STm EU/mL (**A**) and anti-SEn EU/mL (**B**) in the function of the serum dilution tested to assay linearity. The two repeats of each single serum performed by each operator are represented by full symbols (for operator 1) and empty symbols (for operator 2), repeats on different days are shown in red circles for day 1, blue squares for day 2, and green triangles for day 3.

For both *S.* Typhimurium and *S.* Enteritidis, the results for each specific dilution tested were similar to each other, with no variation due to operator, days, or repeats (Figure 3). Linearity was confirmed within the tested range, both in terms of regression analysis and deviation from linearity, calculated by multiplying the mean of the obtained value at each specific dilution by the specific dilution, and by dividing it by the median obtained when testing the undiluted sample (thus, used as nominal value). The average deviation from linearity was within the predefined range of acceptability [0.7–1.3] for both antigens (Table 2).

Both assays resulted to be linear in the tested range. Linear regression applied to the base-2 log-transformed data (geometric means and dilutions) yielded a significant slope both for *S.* Enteritidis (slope = 0.986, t-stat = 73.4, *p* < 0.0001) and for *S.* Typhimurium (slope = 0.965, t-stat = 149.2, *p* < 0.0001). These two similar findings confirmed that the method produced results within the specification limits of linearity [0.7–1.3].

The minimum raw value measured on all runs and dilutions is expressed as the Lower Limit of Linearity (LLL), which resulted to be 3.5 EU/mL in the case of *S.* Typhimurium and 1.7 EU/mL in the case of *S.* Enteritidis.

### 3.4. Precision

**Figure 4:**
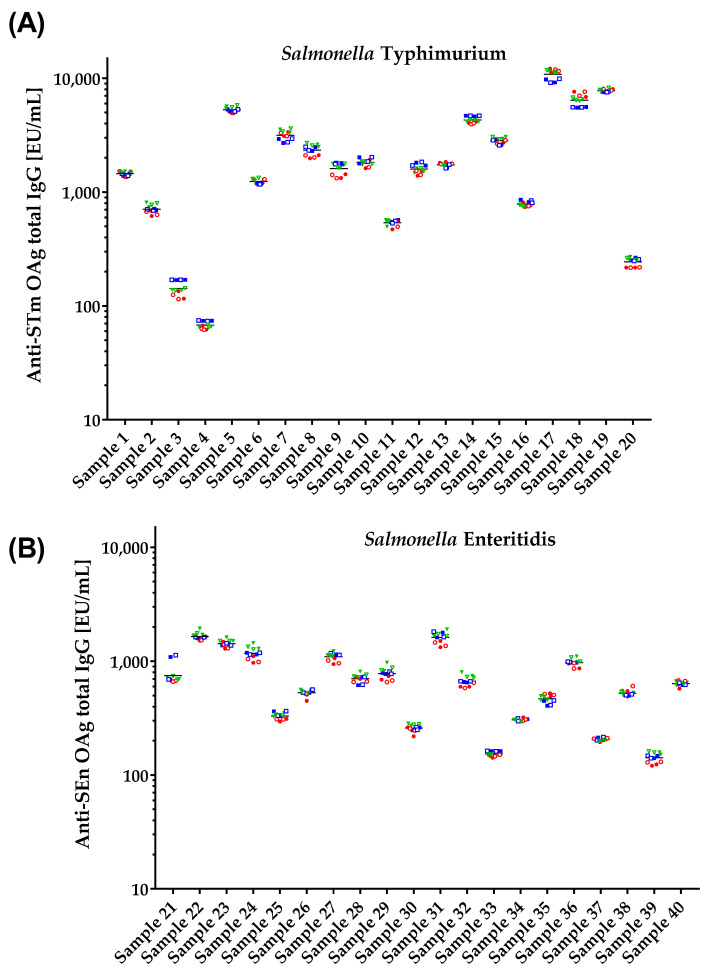
ELISA units/mL obtained in precision test performed probing 40 human single sera: (**A**) 20 against STm OAg and (**B**) 20 against SEn OAg in 2 independent replicates, performed on 3 different days and by two operators. The two repeats of each single serum performed by each operator are represented by full symbols (for operator 1) and empty symbols (for operator 2), repeats on different days are shown in red circles for day 1, blue squares for day 2, and green triangles for day 3. Geometric means from all repeats of each serum tested against both STm and SEn OAgs are represented by the black lines.

To evaluate the precision of the assay, which is the ability of a measurement to be consistently reproduced, EU/mL of 40 human single sera from adults subjects (20 against STm OAg and 20 against SEn OAg) were determined independently by two operators working on the same days, in two replicates, on three different days (12 measurements in total for each individual serum), and results obtained are reported in Figure 4.

Precision was evaluated as repeatability (intra-assay precision) and intermediate precision (inter-assay precision). Repeatability expresses the precision under the same operating conditions while intermediate precision expresses within-laboratories variations, such as different days, different analysts, different equipment, calibrants, batches of reagents, columns, and spray needles.

The critical thresholds (CV%) for repeatability and intermediate precision were 20% and 25%, respectively. We found that repeatability and intermediate precision were below their corresponding threshold across all samples. To generalize these findings, we calculated the 95% bootstrap confidence interval of the geometric mean of the samples’ CV%. Thus, precision results were resampled 1000 times and the geometric mean was calculated at each iteration. For *S.* Typhimurium Oag, the expected geometric mean of the intermediate precisions was 2.66% (CI95% from 2.09% to 3.25%) and the geometric mean of the repeatability was 7.10% (CI95% from 2.38% to 14.80%). The bootstrap analysis of the *S*. Enteritidis OAg data produced a geometric mean of intermediate precisions equal to 3.42% (CI95% from 2.74% to 4.12%) and a geometric mean of repeatability equal to 6.94% (CI95% from 1.20% to 14.49%). 

The day, the operator and the replicates did not influence assay variability for the 20 individual samples of *S*. Typhimurium and 17 out of 20 individual samples tested against *S*. Enteritidis O-antigens. 

The evaluation of the Lower Limit of Precision (LLP) implied two steps: first, the raw data of precision above 600 were divided by 4000 and all the other data were divided by 100; second, the minimum of these rescaled data was considered as the LLP, and this resulted to be 15 EU/mL for both assays.

Lastly, the lower limit of quantification (LLoQ) was calculated as the most conservative value among the lower limit of standard curve accuracy, lower limit of precision, and lower limit of linearity, all obtained considering the limit per well multiplied by the lowest sample dilution tested (100), as reported in Table 3.

The methodology used for the assay does not have an upper limit of quantification, as in the case of readings above the upper limit of standard curve accuracy, a higher dilution of samples (40-fold higher than previous) can be tested to obtain readings falling within the limit of standard curve accuracy and with appropriate precision.

### 3.5. Specificity 

**Figure 5:**
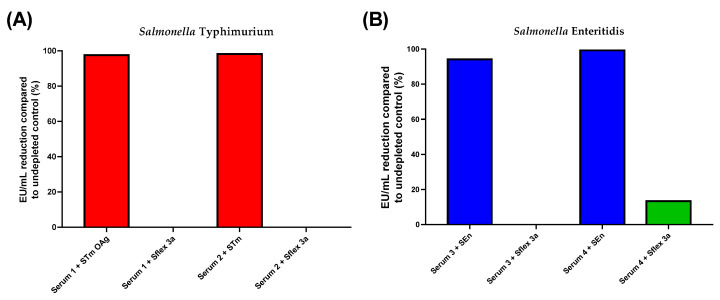
Heterologous specificity results: (**A**) for *S*. Typhymurium and (**B**) for SEn, respectively. ELISA units reduction compared to undepleted control sera expressed as a percentage for each individual sera after incubation with homologous and heterologous competitors at selected concentration of 20 µg/mL. STm: *S*. Typhimurium; SEn: *S*. Enteritidis; Sflex: *S. flexneri*.

The specificity of the assay was determined for both *S.* Typhimurium and *S.* Enteritidis OAg. This parameter represents the ability of the analytical procedure to determine solely the concentration of the analyte that it intends to measure. To evaluate the homologous specificity, an initial set-up experiment was performed by pre-incubating two samples with high anti-*S.* Typhimurium and *S.* Enteritidis OAg IgG titers, respectively, with an equal volume of homologous *S.* Typhimurium and *S.* Enteritidis purified O-Ag at the final concentrations of 250, 50, 20, 5, and 1 µg/mL prior to being tested by ELISA. The goal was to determine the lowest concentration of OAg able to inhibit ≥80% of the EU/mL in comparison to the non-inhibited sample. The lowest Salmonella OAg concentration that was able to cause an inhibition of ≥80% of the detected OAg IgG concentration was 20 µg/mL (data not shown). Therefore, in a subsequent experiment, 20 µg/mL of homologous OAg and heterologous competitor (OAg from *Shigella flexneri* 3a) were added to the samples prior to testing them in a standard assay. The percentage of inhibition was calculated in comparison with undepleted control sera (Figure 5). 

For both *S*. Typhimurium and *S*. Enteritidis assays, the percentage of inhibition detected was confirmed to be ≥80% with a homologous OAg and <20% with a heterologous competitor; therefore, both assays are considered specific. 

## 4. Discussion and Conclusions

To perform a fully quantitative assessment of antigen-specific antibodies in samples from vaccine clinical studies or in sera of subjects after natural exposure to a pathogen, it is key to have a simple and highly sensitive assay. The ELISA methodology presented here has been extensively used at a preclinical level to develop a vaccine to prevent iNTS disease [10] and to determine the level of antigen-specific human antibodies in clinical trials of a vaccine against *Shigella* in endemic [23] and non-endemic settings [24,25,26]. In this work, we set up, optimized, standardized, and characterized the ELISA method to determine the level of specific IgG elicited by *S.* Typhimurium and *S.* Enteritidis OAgs in human sera. The method resulted to be precise, accurate, repeatable, linear, and specific for both *S.* Typhimurium and *S*. Enteritidis OAgs. The assay has a broad standard accuracy range, is linear and specific for the OAg coated on the plate, and has a dynamic range going from a very low limit of quantification to essentially no upper limit of quantification. The assays for both *S*. Typhimurium and *S*. Enteritidis were demonstrated to be precise, with a low variability on 20 individual samples, with neither the operator, nor the day of analysis, nor the replicates being significant to the overall variability. Furthermore, for the precision assessment, mainly samples with medium to high levels of antigen-specific IgG induced upon natural exposure have been tested; thus, we cannot exclude that with a different and broader selection of samples, including the ones with low titers, we would determine even a lower limit of precision. This latter aspect will be reassessed during the formal validation of the assay when samples from vaccines will also be available.

The ELISA assay described here has a continuous readout, which has several advantages compared with the titer-based approach. A critical advantage is related to the ability to evaluate and compare the immune response induced by vaccination among different individuals. Indeed, one of the most widely used methods to evaluate vaccine response is the ELISA with the seroconversion expressed as a 4-fold increase compared to baseline as a proxy for it [27]. In titer-based assays, often a small change of even 0.1 OD might result in a half or double titer and this could lead to misinterpretation of the immunological results. This is not the case for fully quantitative assays with continuous readout such as the ELISA methodology presented in this work.

One of the limitations of the traditional ELISA, like the assay presented here, could be the fact that it is not multiplexed. However, this type of ELISA assay is usually of low cost and can be easily transferred and used in other laboratories, including resource-limiting settings. Furthermore, traditional ELISA can be easily automated to increase throughput, reducing the time to produce results compared with multiplex assays, and in terms of costs, considering the low cost of consumables in classical ELISA versus the costs of multiplex-based assays requiring more expensive reagents, equipment and infrastructure, unless a high number of analytes is simultaneously assessed (i.e., >5).

The assay presented here is versatile and, with minimal adjustments, can be easily adapted to determine antibody levels from different sample sources (feces, plasma, and saliva) or against different types of antigens (protein or polysaccharide), and detect other antigen-specific immunoglobulin classes (IgM or IgA) or IgG subclasses. These other possibilities would still maintain the same definition of EU/mL, which represents the most important criterion of standardization in our assay. The presence of a standard serum as an inter-laboratory calibrator guarantees the robustness of the assay, and the simplicity and affordability of the assay make it suitable for other laboratories interested in analyzing large sero-epidemiological studies or vaccine clinical trials. Indeed, the assay has been already successfully transferred to different laboratories, including sites in African endemic regions, and has demonstrated the interlaboratory transferability of the results presented in this work. Unlike some other platforms, an ELISA format is easily transferred and accessible to resource-limiting settings, where the iNTS disease is endemic. We have also recently presented a high-throughput method to evaluate the ability to kill *S*. Enteritidis and *S*. Typhimurium in serum samples by using an iNTS standard sera [28]. Performing the correlation among the level of antibodies and antibody functionality from serum samples and clearance from the disease might allow us to define a protective threshold or correlate of protection, which could speed up vaccine development.

To conclude, the assay presented here can accurately quantify the antibodies against *S.* Typhimurium and *S*. Enteritidis OAgs in human serum samples and can be applied to both large sero-epidemiological studies and vaccine clinical trials. The use of the same assay in both studies will allow us to compare the data and evaluate potential differences in immune response between vaccinated subjects or subjects recovering from the disease. Furthermore, this analysis could be applied to select appropriate sites and vaccine trial strategies, to advance the development of vaccines against iNTS disease.

## Figures and Tables

**Table 1:** Quality controls acceptance criteria for standard curves and controls.

	Minimum R^2^	Maximum Background OD	Minimum Value of OD Max	OD at 1 EU	Controlled Deviation to the Expected EU/mL for HC and LC	HC–LC Expected Value [EU/mL]
*S.* Typhimurium	0.97	<0.15	2.8	From 0.6 to 1.5	<40%	2.42–1.3
*S.* Enteritidis	0.97	<0.15	3.0	From 0.7 to 1.5	<40%	2.77–1.45

**Table 2:** Average deviation from linearity.

	Average Deviation from Linearity	
	Min	Max
*S.* Typhimurium	0.82	1.26
*S.* Enteritidis	0.87	1.10

**Table 3:** Lower limit of quantification (LLoQ).

	LLP [EU/mL]	LLP [EU/mL]	LLSCA [EU/mL]	LLoQ [EU/mL]
*S.* Typhimurium	15	4	4	15
*S.* Enteritidis	15	2	13	15

## Data Availability

Not applicable.
